# Ultrasound-Guided Calcific Barbotage for Rotator Cuff Calcific Tendinopathy: A Case Report

**DOI:** 10.7759/cureus.104831

**Published:** 2026-03-07

**Authors:** Leya Rum, Kevin E Elder, Niharika Suchak

**Affiliations:** 1 College of Medicine, Florida State University College of Medicine, Tallahassee, USA; 2 Sports Medicine, BayCare Medical Group, Tampa, USA; 3 Medical Education and Simulation, Florida State University College of Medicine, Tallahassee, USA

**Keywords:** barbotage, calcific barbotage, calcific tendinopathy, rotator cuff tendinopathy, ultrasound-guided

## Abstract

Calcific tendinopathy of the rotator cuff is a common cause of shoulder pain and restricted function, most frequently involving the supraspinatus tendon. While conservative therapy and corticosteroid injections are first-line treatments, some patients experience persistent symptoms. Ultrasound-guided calcific barbotage is a minimally invasive intervention that directly targets calcific deposits.

A 68-year-old Asian woman with hypertension and degenerative joint disease presented with a two-month history of left shoulder pain and limited range of motion. Prior corticosteroid injection failed to provide relief. Imaging revealed a calcific deposit in the left supraspinatus tendon. She underwent ultrasound-guided barbotage using an 18G needle for mechanical fragmentation and aspiration.

The procedure was well tolerated without complications. At the five-day follow-up, the patient reported a significant reduction in pain and restoration of full shoulder abduction and overhead motion. Follow-up MRI at two months confirmed maintained rotator cuff integrity and resolution of the calcific deposit.

Ultrasound-guided calcific barbotage is an effective, minimally invasive option for patients with rotator cuff calcific tendinopathy refractory to conservative management.

## Introduction

Calcific tendinopathy is characterized by the deposition of calcium hydroxyapatite crystals within tendons, most commonly affecting the rotator cuff [1]. The supraspinatus tendon is frequently involved, resulting in shoulder pain and functional limitations [1]. Although the etiology of calcific tendinopathy remains unclear, two primary theories have been proposed. The first is degenerative calcification, in which age-related changes in the tendon lead to decreased vascularity, resulting in tendon necrosis and subsequent calcification [2]. The second theory describes calcification as a dynamic process occurring in three stages: precalcific, calcific, and postcalcific [2]. During the precalcific stage, fibrocartilaginous transformation of the tendon occurs due to chondrocyte formation [2]. In the calcific stage, calcium deposits form and are subsequently resorbed by phagocytes [2]. The postcalcific stage is characterized by granulation tissue formation within the space previously occupied by the calcific deposit [2].

Calcific tendinopathy most commonly affects adults aged 40-60 and has a higher prevalence in women [3]. Clinical manifestations typically include shoulder pain exacerbated by abduction or overhead movements and are often worse in the morning [3]. First-line imaging includes shoulder radiography, such as X-ray, which can identify calcific deposits and guide further evaluation [3]. Initial management of calcific tendinopathy generally consists of nonsteroidal anti-inflammatory drugs, physical therapy, and corticosteroid injections [1]. However, some patients continue to experience persistent symptoms despite conservative treatment. In this article, we present the case of a 68-year-old woman with calcific tendinopathy refractory to conservative management who successfully underwent ultrasound-guided calcific barbotage of the left rotator cuff.

## Case presentation

A 68-year-old woman with hypertension and degenerative joint disease presented in the outpatient setting with a one-year history of shoulder stiffness, which had progressed to severe pain over the last two months. She described left shoulder stiffness worsening with abduction and difficulty with activities such as dressing and overhead movements. She denied previous trauma. Initial management consisted of oral meloxicam, topical diclofenac, and a structured course of physical therapy focused on rotator cuff strengthening and range-of-motion exercises. She experienced intolerance to medications, including a rash with topical diclofenac, but maintained adherence to the physical therapy sessions as well as at-home exercises. After months of continued pain, a corticosteroid injection was administered at an outside institution, although the exact timing could not be confirmed.

**Figure 1:**
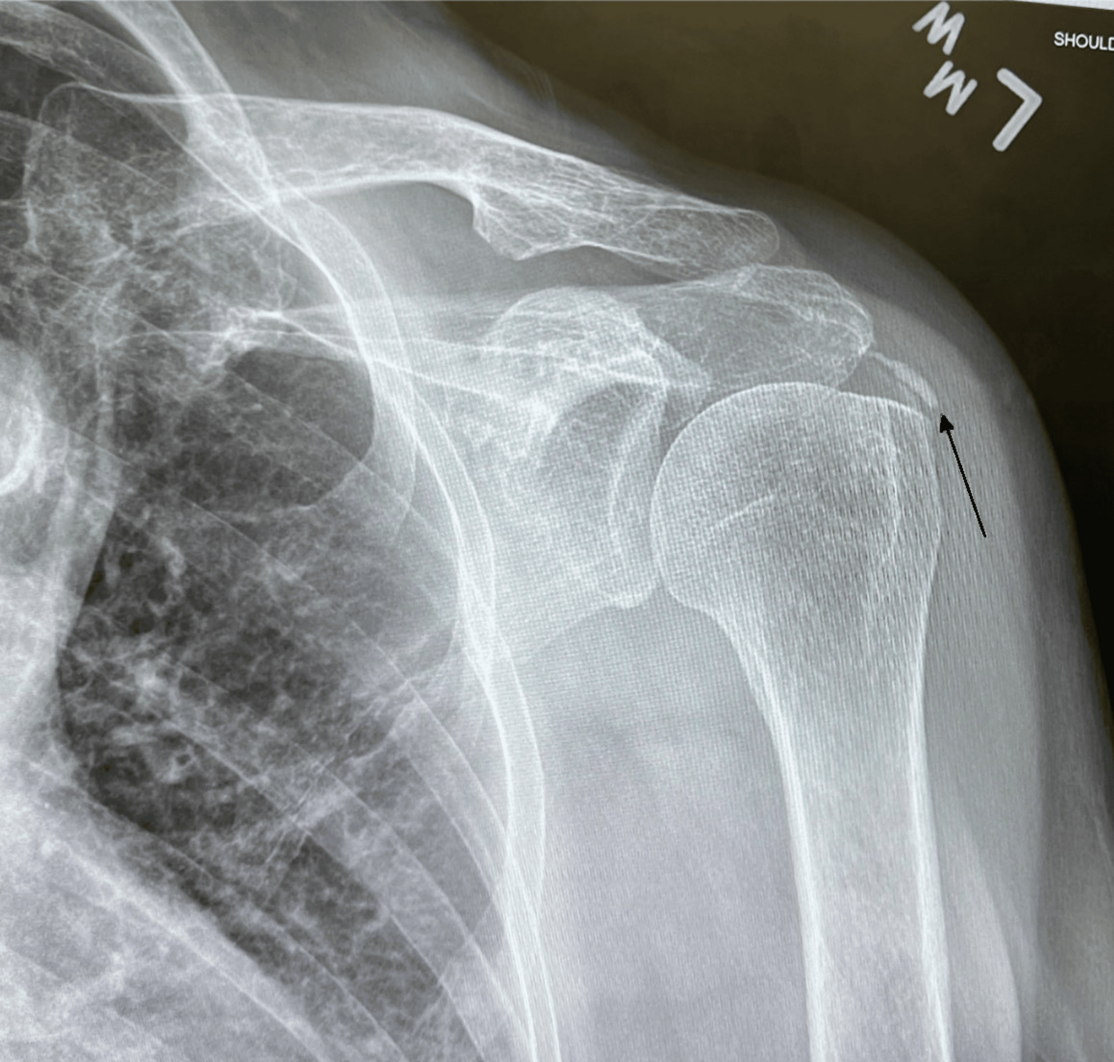
X-ray image of left shoulder showing a calcific deposit, indicated by the arrow.

Physical examination revealed limited active range of motion, particularly with left arm abduction. There was no evidence of effusion, edema, or muscular atrophy. Plain radiography demonstrated a calcific deposit in the left supraspinatus tendon (Figure 1).

**Figure 2:**
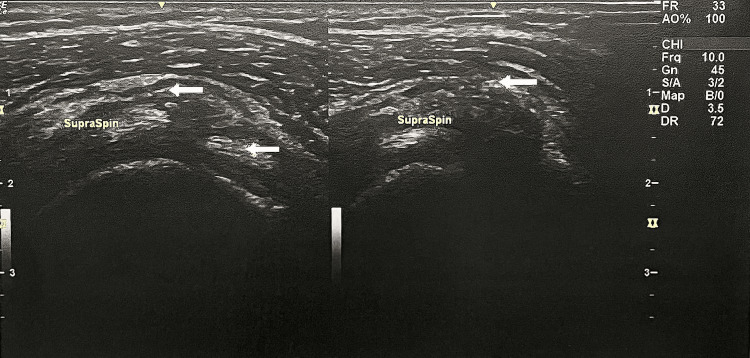
Ultrasound image of calcific deposit, shown by arrows.

Three days after presentation, the patient underwent ultrasound-guided calcific barbotage. Under local anesthesia, an 18G needle was introduced into the calcific deposit under ultrasound guidance (Figure 2). The deposit was mechanically fragmented and aspirated using negative-pressure saline lavage totaling 10 cc. At the end of the procedure, a mix of 4 mg dexamethasone and lidocaine was injected into the bursa to target post-procedure pain and inflammation. Ultrasound guidance ensured precise needle placement and avoided vascular structures. The procedure was well tolerated without complications.

At the five-day follow-up, the patient reported marked improvement in pain. Physical examination revealed full shoulder abduction and overhead motion. Follow-up at two months included an MRI that was obtained at the patient's request to establish a post-procedural imaging baseline and assess residual calcification and tendon integrity. Results confirmed resolution of the calcific deposit as well as normal rotator cuff integrity. The patient reported improved functionality in daily activities.

## Discussion

Management of rotator cuff calcific tendinopathy remains variable once patients fail conservative therapy, and optimal escalation strategies continue to be debated [2]. Minimally invasive interventions such as ultrasound-guided calcific barbotage have gained increasing attention as alternatives to surgical management, particularly in patients with persistent symptoms and well-defined calcific deposits [2]. The present case provides an opportunity to examine the role of calcific barbotage as an intervention for rotator cuff tendinopathy and contextualizes its success within current literature.

Previous studies have demonstrated that failure of conservative management is not uncommon in calcific tendinopathy. In a large prospective cohort of 420 patients, 27% required escalation to specialized interventions, including ultrasound-guided barbotage, following inadequate response to initial therapy [4]. These findings underscore the need for effective minimally invasive options in patients with ongoing symptoms. Barbotage directly addresses the underlying pathology by fragmenting and aspirating calcific deposits, thereby facilitating symptom resolution and functional improvement while avoiding the morbidity associated with surgical intervention [1].

Comparative studies further support the efficacy of barbotage over corticosteroid injections alone. In a randomized controlled trial, patients treated with ultrasound-guided barbotage demonstrated greater reduction in calcific deposit size and superior clinical outcomes at one-year follow-up compared with those receiving corticosteroid injections [5]. These results suggest that mechanical removal of calcifications offers advantages beyond anti-inflammatory treatment alone, particularly in patients with well-defined deposits.

Additional literature supports the short-term effectiveness of ultrasound-guided barbotage in improving pain outcomes for patients with calcific tendinopathy. In a retrospective case series of 179 ultrasound-guided barbotage procedures, patients demonstrated significant reductions in shoulder pain at short-term follow-up, particularly at two months post-procedure, compared with preprocedural scores [6].

Ultrasound guidance plays a critical role in optimizing procedural success and safety. It allows precise localization of calcific deposits, real-time visualization during needle placement, and avoidance of adjacent neurovascular structures [1]. Potential complications of calcific barbotage include infection, bruising, bursitis, tendon injury, and hematoma formation, all of which were monitored at follow-up visits and were notably absent [1]. In this case, ultrasound guidance facilitated accurate targeting of the calcific deposit and contributed to a favorable clinical outcome.

While barbotage has demonstrated favorable results, patient selection remains essential. Optimal candidates are those with persistent symptoms despite conservative management and calcific deposits that are accessible and amenable to ultrasound-guided intervention [2]. This case adds to the growing body of evidence supporting ultrasound-guided calcific barbotage as an effective treatment option for appropriately selected patients with rotator cuff calcific tendinopathy refractory to conservative management.

## Conclusions

The successful management of this 68-year-old patient highlights the clinical utility of ultrasound-guided calcific barbotage as a potential bridge between failed conservative therapy and invasive surgery. While corticosteroid injections primarily address secondary inflammation, barbotage targets the underlying pathology by facilitating direct fragmentation and aspiration of hydroxyapatite deposits. In this case, rapid restoration of overhead function and MRI-confirmed resolution of the calcific deposit within two months support the effectiveness of this approach. This outcome suggests that ultrasound-guided barbotage may represent a viable option for appropriately selected patients with calcific tendinopathy refractory to first-line management.
